# Commentary on Kersbergen *et al*.: Same Price, same choices? Proportional pricing and the heaviest drinkers

**DOI:** 10.1111/add.70046

**Published:** 2025-03-25

**Authors:** Robyn Burton, Nick Sheron

**Affiliations:** ^1^ Institute for Social Marketing and Health University of Stirling Stirling UK; ^2^ The Roger Williams Institute of Liver Studies Kings College London London UK

**Keywords:** daily drinkers, heavy drinkers, on‐line experiment, price, proportional price, purchasing

*Proportional pricing changes purchasing incentives but does not directly target the affordability of the cheapest or strongest alcohol. Its real‐world impact on heavy drinkers remains uncertain, particularly if retailers adjust pricing strategies. This highlights the need to critically reflect on whether the policy targets those most at risk of harm*.

Kersbergen *et al*. [1] provide experimental evidence that proportional pricing—where alcohol is priced consistently per litre across different package sizes of the same brand—can shift purchasing preferences toward smaller products in hypothetical scenarios. While this approach changes purchasing incentives, it does not directly address the affordability of cheap or high‐strength alcohol. These limitations raise important questions about its effectiveness, particularly for the heaviest drinkers.

Heavy daily drinkers face exponentially higher risks of alcohol‐related harm [2], making them a key target for policy intervention. They typically seek out the cheapest alcohol [2, 3, 4], but were not included in Kersbergen *et al*. [1] study. Unlike minimum unit price (MUP), which directly raises the price of the lowest‐cost alcohol [5, 6], proportional pricing removes bulk discounts within brands, but does not necessarily increase the absolute price of the cheapest products.

From a public health perspective, stronger alcohol should cost more because of its greater harm potential. A typical serving of wine results in a higher peak blood alcohol concentration than beer, whereas for spirits, the peak is nearly twice that of beer and reached more quickly [7]. Volumetric taxation, recently adopted in the United Kingdom [8], links price to alcohol content, making higher‐strength products relatively more expensive and discouraging excessive consumption [9, 10]. In contrast, proportional pricing equalises cost per litre within brands but does not account for alcohol strength. As a result, a stronger product may still be cheaper per unit than a weaker one, depending on retailer pricing strategies.

Proportional pricing raises the cost of larger products by aligning their per‐litre price with smaller equivalents, but its impact on real‐world pricing strategies remains uncertain. Retailers have previously adapted to policy changes, as seen following Scotland's multi‐buy discount ban, where straight (single unit) discounts became more common [11, 12]. A similar response could occur if retailers offset price increases for larger products by reducing the per‐litre costs of smaller ones. If so, the overall cost of alcohol would remain unchanged, limiting the policy's impact.

A further consideration is how heavy daily and dependent drinkers purchase alcohol. While there is limited research on portion sizes in this group, anecdotal clinical experience suggests that some may buy alcohol daily in quantities just sufficient for that day. This may be an attempt to manage their consumption, as purchasing a larger volume intended to last multiple days could result in it being consumed more quickly than planned. If proportional pricing were to reduce the cost of smaller portions, this could unintentionally make alcohol more affordable for those who purchase daily in small quantities. Further research is needed to understand these potential behavioural effects before the policy's real‐world implications can be fully assessed.

There is no shortage of policy strategies that appear effective in principle, but have minimal real‐world impact. A precedent exists in the United Kingdom's ban on below‐cost sales, which prohibited alcohol from being sold below the combined cost of value added tax and excise duties. While this measure signalled action on alcohol pricing, it affected less than 1% of alcohol units purchased by harmful drinkers, whereas a £0.50 MUP affected 44% of their purchases [13]. The concern being that these politically expedient policies may occupy the bandwidth of policymakers, diverting attention and resources away from more impactful strategies to prevent harm in the heaviest drinkers. It is too early to say where proportional pricing falls on this spectrum, but real‐world studies, including very heavy daily and dependent drinkers, should form a key part of any future assessment.

## DECLARATION OF INTERESTS

None.

## Data Availability

No data are available.
